# Skeletal muscle mitochondrial bioenergetics in pregnancy: a comparison of in vivo and in vitro outcomes

**DOI:** 10.1210/jendso/bvag093

**Published:** 2026-04-10

**Authors:** Ericka M Biagioni, Polina M Krassovskaia, Gillian Tiralla, Kelsey H Fisher-Wellman, Chien-Te Lin, Abby D Altazan, R Caitlin Hebert, Sripallavi Yendamuri, Maninder Singh, Owen T Carmichael, P Darrell Neufer, Kristen E Boyle, Leanne M Redman, Nicholas T Broskey

**Affiliations:** Department of Kinesiology, East Carolina University, Greenville, NC 27858, USA; Human Performance Laboratory, East Carolina University, Greenville, NC 27858, USA; East Carolina Diabetes and Obesity Institute, East Carolina University, Greenville, NC 27834, USA; Department of Cancer Biology, Wake Forest University School of Medicine, Winston-Salem, NC 27157, USA; Department of Kinesiology, East Carolina University, Greenville, NC 27858, USA; Human Performance Laboratory, East Carolina University, Greenville, NC 27858, USA; East Carolina Diabetes and Obesity Institute, East Carolina University, Greenville, NC 27834, USA; Department of Cancer Biology, Wake Forest University School of Medicine, Winston-Salem, NC 27157, USA; Department of Internal Medicine, Section on Molecular Medicine, Wake Forest University School of Medicine, Winston-Salem, NC 27157, USA; Pennington Biomedical Research Center, Louisiana State University, Baton Rouge, LA 70808, USA; Pennington Biomedical Research Center, Louisiana State University, Baton Rouge, LA 70808, USA; Department of Kinesiology, East Carolina University, Greenville, NC 27858, USA; Human Performance Laboratory, East Carolina University, Greenville, NC 27858, USA; East Carolina Diabetes and Obesity Institute, East Carolina University, Greenville, NC 27834, USA; Pennington Biomedical Research Center, Louisiana State University, Baton Rouge, LA 70808, USA; Pennington Biomedical Research Center, Louisiana State University, Baton Rouge, LA 70808, USA; Department of Internal Medicine, Section on Molecular Medicine, Wake Forest University School of Medicine, Winston-Salem, NC 27157, USA; Department of Pediatrics, University of Colorado Anschutz Medical Campus, Aurora, CO 80045, USA; Pennington Biomedical Research Center, Louisiana State University, Baton Rouge, LA 70808, USA; Department of Kinesiology, East Carolina University, Greenville, NC 27858, USA; Human Performance Laboratory, East Carolina University, Greenville, NC 27858, USA; East Carolina Diabetes and Obesity Institute, East Carolina University, Greenville, NC 27834, USA

**Keywords:** exercise, metabolism, mitochondria, myotube, pregnancy

## Abstract

**Purpose:**

Exercise-mediated adaptations to mitochondria are well established in nongravid populations; however, the extent to which these adaptations occur during pregnancy remains unclear. Therefore, the objective of this study was to compare skeletal muscle mitochondrial bioenergetics in physically active (*n* = 10) vs sedentary (*n* = 9) pregnant women.

**Methods:**

Groups were matched for age, race, and pregravid body mass index and were studied in the second (T2; weeks 21-25) and third trimester (T3; weeks 31-35). Free-living physical activity was assessed by accelerometry and aerobic fitness by peak oxygen uptake (VO_2_peak) testing. In vivo mitochondrial capacity was assessed by ^31^P-magnetic resonance spectroscopy. Primary skeletal muscle myotubes were obtained via muscle biopsy between late T2 and early T3. Mitochondrial in vitro respiration was assessed by high-resolution respirometry, and mitochondrial content was measured by Western blot and enzyme activity.

**Results:**

Despite a decline in physical activity across gestation, active women maintained a higher VO_2_peak at T2 (*P* < .05) and T3 (*P* < .01) compared with sedentary. There were no differences in phosphocreatine recovery time between groups or timepoints. Myotube mitochondrial respiratory capacity was similar between groups; however, compared with sedentary mothers, active mothers demonstrated increased expression of mitochondrial complexes I, II, and IV proteins (all *P* < .05). Additionally, myotube mitochondrial efficiency (adenosine triphosphate-to-oxygen consumption ratio) measures were positively correlated with maternal VO_2_peak at T3 (r = 0.49, *P* < .05), suggesting a link between fitness and mitochondrial efficiency.

**Conclusion:**

These findings suggest that late pregnancy may blunt mitochondrial adaptations to aerobic exercise despite a preservation of cardiovascular fitness. Future studies are needed to determine whether increasing activity throughout gestation can enhance mitochondrial respiration.

Sedentary behavior is prominent during pregnancy with some epidemiological reports suggesting 50% to 60% of women do not engage in regular physical activity [1]. Historically, exercise during pregnancy was thought to be detrimental to the developing fetus; however, current guidelines recommend physical activity for most pregnant women [2]. The importance of maternal exercise is evident in the control of lipids [3, 4], blood glucose [5], blood pressure [6], and gestational weight gain [7]. Conversely, a sedentary lifestyle and maternal obesity can predispose mothers to complications such as gestational diabetes mellitus (GDM) [8] and preeclampsia [9]. Although the benefits of exercise during pregnancy for the expecting mother are widely accepted, the mechanisms through which these occur have yet to be elucidated.

Mitochondria play a fundamental role in energy generation within virtually all cells, including prominent and essential roles in skeletal muscle metabolism. Alterations in mitochondrial energetics within skeletal muscle have been associated with obesity and type 2 diabetes, as well as physical inactivity and aging [10]. Conversely, exercise elicits beneficial adaptations within skeletal muscle [11]. With exercise, skeletal muscle becomes accustomed to higher energy requirements [12], in turn, stimulating mitochondrial adaptations such as increased oxidative capacity [13] and biogenesis [14]. Thus, many of the health benefits from exercise are purported to stem from mitochondrial adaptations, yet an understanding of these adaptations in the skeletal muscle of expectant mothers has yet to be uncovered.

Perinatal exercise interventions are commonly used to improve the health of the mother; however, studies exploring skeletal muscle mitochondrial bioenergetics in pregnancy are lacking. One avenue to fill this gap is through the use of primary cells. Primary human cells have been used to investigate cell-autonomous mechanisms that underlie the effects of lifestyle interventions, including exercise [15], as well as the pathophysiology of diseases, including diabetes [16], obesity [17, 18], and peripheral arterial disease [19]. For example, primary skeletal muscle cells (SKMcs) derived from exercise-trained subjects retain the hallmark adaptations seen in vivo, including elevations in lipid-handling capacity [20, 21], oxidative capacity [15], and insulin sensitivity [22]. Thus, in vitro assays recapitulate features of the in vivo phenotype, supporting their use as a model for exploring cellular mechanisms underlying metabolic function. Yet, to our knowledge, there have been no studies assessing skeletal muscle mitochondrial bioenergetics in expectant mothers utilizing SKMcs. Additionally, most studies that have investigated the impact of exercise during pregnancy aim to recruit sedentary participants and do not implement an exercise intervention until more than halfway through the first trimester [23, 24], with some starting as late as T3 [25, 26]. Considering that most exercise adaptations occur within the first months of beginning an exercise regimen, this results in an understudied population of women who are active prior to and during pregnancy, and thus, it remains unknown whether the bioenergetic benefits of exercise accumulated prior to conception are retained through pregnancy. Therefore, the purpose of this study is to examine differences in the mitochondrial bioenergetics between women who are aerobically active before and during pregnancy and their matched sedentary counterparts.

## Materials and methods

### Ethics statement

This study used skeletal muscle tissue biopsies collected from participants enrolled in the Mito Moms Study (The Effect of Physical Activity on In Vivo and In Vitro Mitochondrial Capacity in Pregnant Women; ClinicalTrials.gov Identifier: NCT03489564). This study was approved by the Pennington Biomedical Research Center (PBRC) Institutional Review Board, and all subjects provided written consent prior to enrollment. All clinical visits occurred at Pennington Biomedical Research Center in Baton Rouge, LA.

### Participant recruitment

Women between 18 and 40 years of age, a pregravid body mass index (BMI) of 18.5 to 30.0 kg/m^2^, and <21 weeks’ gestation who self-defined as active or sedentary were recruited with a targeted advertising campaign (Facebook advertisements, flyers, Pennington Biomedical Research Center clinical trials LISTSERV email list) and direct referrals from local prenatal care providers. Women with GDM, preeclampsia, a history of preterm delivery, HIV, or a family history of diabetes were excluded from the study. Participants were matched for age, race, pregravid BMI, and fetal sex upon delivery. All subjects had a negative family history of type 2 diabetes to rule out an inheritance of dysfunctional mitochondria as previously reported [27]. A screening visit occurred at <21 weeks’ gestation to determine eligibility and confirm physical activity level, as well as measure body composition via air-displacement plethysmography (BOD POD®). Physical activity level was classified by self-report and confirmed via accelerometer (SenseWear, Body Media, Pittsburgh, PA) as either active (≥8000 steps/day or ≥3 bouts of moderate to vigorous physical activity [MVPA] for ≥30 minutes per week) or sedentary (≤5000 steps/day or <3 bouts of MVPA for ≥30minutes per week) at screening. Clearance for study participation was granted by the study's medical investigator after review of the PARmed-X [28] for pregnancy. Participants’ medical providers provided clearance of the study's procedures.

### Maternal study visits

Study outcomes were collected at 2 visits. The first between 21 and 25 weeks (T2) and the second between 31 and 35 weeks of gestation (T3). During these visits, weight, blood pressure, aerobic fitness, in vivo mitochondrial capacity, and free-living physical activity were measured.

#### Questionnaires

The Stanford Physical Activity Recall and Sedentary Behavior Questionnaire was administered in addition to 1 week of activity monitoring via accelerometer to confirm self-reported activity level. Participants also completed a physical activity journal to confirm bouts of exercise and that all active participants were aerobically trained. Step count and MVPA were collected at screening, T2, 26 to 30 weeks (midpoint between T2 and T3), and T3. Participants completed a food frequency questionnaire (Diet History Questionnaire-III) [29] to account for differences in dietary intake.

#### Aerobic fitness

To assess aerobic fitness, participants underwent a progressive submaximal VO_2_ treadmill test up to 80% of peak oxygen uptake (VO_2_peak) adapted from the Balke protocol as described elsewhere [30]. All VO_2_peak tests were conducted using a True Max 2400 Metabolic Measurement Cart (Parvomedics, Salt Lake City, UT).

#### In vivo mitochondrial capacity

In vivo maternal mitochondrial capacity of skeletal muscle was measured using ^31^P-magnetic resonance spectroscopy (MRS). All phosphorus-31 magnetic resonance spectroscopy (^31^P-MRS) experiments were performed on a GE 3T Signa HDxT MR scanner (GE Healthcare, Milwaukee, WI) using a transmit-receive (60 mm diameter) dual-tuned ^1^H/^31^P single loop (Doty Scientific) surface coil. The isometric leg extension exercise protocol has been described previously [31]. Briefly, participants were positioned supine on the scanner table in a feet-first orientation. Legs were kept straight without inclination and strapped to the scanner table at the knee and ankle positions with nonelastic Velcro straps. The coil was centered 6 to 8 inches from the patella toward the greater trochanter on the side of the right quadricep over the vastus lateralis muscle and positioned with a Velcro strap.

The magnetic resonance imaging session begins with ^1^H magnetic resonance imaging of the right quadricep muscle with a standard single-shot fast/turbo spin echo sequence, along with the scanner body coil to localize the vastus lateralis muscle. A vitamin E pill at the center of the ^31^P surface coil was used to localize the surface coil within the imaging data and confirm coil placement. Magnetic field shimming and phosphorus-31 radiofrequency pulse calibration were performed using procedures described previously [31]. Two ^31^P-MRS datasets were acquired from the same single voxel (size: 5 × 5 × 2 cm [3]) placed within the vastus lateralis muscle, using a localized standard pulse and acquire sequence. The first dataset was recorded in the resting state. The second dataset was acquired during and after an exercise bout during 1 minute of rest, followed by 25 to 30 seconds of moderate isometric leg kicking and 6 minutes of recovery. While exercising, the participant received instructions to perform isometric leg kicking against the straps.

The postprocessing of spectral data was completed using jMRUI 5.2 software. Initial steps include applying 5 Hz of Gaussian line broadening to the raw data, followed by the zero-order phase correction. Then, the Advanced Method for Accurate, Robust, and Efficient Spectral fitting algorithm was used to quantify time domain spectral peaks, including the phosphocreatine (PCr) peak [32]. The peak heights quantified using the Advanced Method for Accurate, Robust, and Efficient Spectral fitting were transferred to MATLAB, and the time series of PCr peaks was fitted to a monoexponential recovery equation [31] using nonlinear least squares curve fitting. The resulting monoexponential curve parameters, including the PCr recovery rate, as well as goodness-of-fit parameters from curve fitting, were recorded. Curves with goodness of fit less than R^2^ < 0.7 were rejected; the PCr recovery rate from the monoexponential curve analysis was a primary outcome of interest in this study.

### Muscle biopsy

A muscle biopsy was taken from the vastus lateralis with the Bergstrom biopsy technique [33] at a separate study visit between T2 and T3 at 26 to 30 weeks’ gestation.

### Primary skeletal muscle cell culture

Satellite cells were isolated from muscle biopsies and amplified on type-I collagen-coated plates until reaching approximately 80% to 90% confluence in growth media (low-glucose DMEM supplemented with 10% fetal bovine serum and 100 μg/mL penicillin/streptomycin) in a 5% CO_2_ and 37 °C humidified atmosphere. Upon reaching 80% to 90% confluence, myoblasts were differentiated to myotubes by switching to differentiation media (low-glucose DMEM supplemented with 2% horse serum, 0.3% BSA, and 100 μg/mL penicillin/streptomycin).

### In vitro mitochondrial function

A series of diagnostic assays using high-resolution respirometry (Oroboros Oxygraph-2K, Oroboros Instruments, Innsbruck, Austria) was conducted to assess in vitro mitochondrial function in intact and digitonin-permeabilized myotubes [34]. All experiments were performed at 37 °C with a continuous stir speed of 500 rpm and used either intact cell respiration media (low-glucose DMEM without HCO_3_, 20 mM HEPES (pH 7.4), 100 units/mL penicillin, 100 µg/mL streptomycin, and 10% heat-inactivated fetal bovine serum) or permeabilized cell respiration buffer (105 mM potassium-MES (pH 7.1), 30 mM KCl, 10 mM KH_2_PO_4_, 5 mM MgCl_2_, 1 mM EGTA, 0.5 mg/mL BSA, and 5 mM creatine monohydrate). At the end of each protocol, mitochondrial respiration was inhibited with rotenone (Rot; 0.5 µM) and antimycin A (AA; 0.5 µM) as a negative control; residual respiration is indicative of nonmitochondrial respiration and subtracted from other rates.

#### Intact cell respirometry

Intact myotubes were resuspended in intact cell respiration media at a concentration of 1.5 × 10^6^ viable cells/mL. Basal rates of oxygen consumption (*J*O_2_) were recorded in the presence of 1 mM pyruvate, 10 mM glucose, and 4 mM glutamine, followed by sequential additions of oligomycin (Oligo; 5 µM), carbonyl cyanide-4-(trifluoromethoxy) phenylhydrazone (FCCP; 2-5 µM), AA, and Rot. FCCP was titrated in incremental additions to achieve maximal uncoupled respiration. The rate corresponding to the peak response was used for analysis and reported in the figures.

#### Phosphorylation efficiency

The adenosine triphosphate (ATP)-to-oxygen consumption (ATP/O) ratio, a measure of mitochondrial phosphorylation efficiency, was quantified by simultaneous measures of ATP synthesis (*J*ATP) and *J*O_2_, described previously [35]. Initially, digitonin-permeabilized myotubes were resuspended in respiration buffer at a concentration of 1.0 × 10^6^ viable cells/mL and loaded into a 1.3 mL reaction chamber. Following the addition of carbon substrates (pyruvate, malate, glutamate, octanoyl-l-carnitine, succinate; 5 mM, 1 mM, 5 mM, 0.25 mM, 5 mM, respectively) and adenosine 5′-diphosphate (ADP; 200 µM), *J*ATP was determined by measuring nicotinamide adenine dinucleotide phosphate autofluorescence at excitation/emission parameters of 340:450.

#### Respiratory flux control

The creatine kinase (CK) clamp was used to assess mitochondrial control of respiratory flux. Initially, digitonin-permeabilized myotubes were resuspended in permeabilized cell respiration buffer at a concentration of 1.5 × 10^6^ viable cells/mL and loaded into a 1 mL reaction chamber. Steady-state *J*O2 was recorded, and then myotubes were energized with nicotinamide adenine dinucleotide–linked carbon substrates (P/M). The CK clamp was initiated by adding PCr (1 mM), ATP (5 mM), and CK (20 mM) to stimulate flux at the highest cellular energy demand of ΔG_ATP_ −12.94 kcal·mol^−1^. Mitochondrial membrane integrity was confirmed by the addition of cytochrome C (0.01 mM) with no change to *J*O_2_, then the remaining carbon substrates (G/O/S) were added. PCr was titrated to clamp ΔG_ATP_ at −14.08 (5 mM), −14.18 (9 mM), −14.49 (15 mM), and −14.70 (21 mM) kcal·mol^−1^, with steady state *J*O_2_ recorded at each point, followed by sequential additions of oligo, FCCP titration (the rate corresponding to the peak response was used for analysis and reported in Fig. 3A and B), AA, and Rot.

### Mitochondrial content

Citrate synthase (CS) activity was used as a surrogate measure of mitochondrial content in myotubes [36] and assessed by colorimetric plate-based assay, as described elsewhere [37].

### Western blotting

Skeletal muscle tissue from muscle biopsies obtained from the vastus lateralis was homogenized and used to probe for protein expression of oxidative phosphorylation (OXPHOS) complexes I to V (Abcam, ab110411, RRID:AB_2756818), CS (Abcam, ab96600, RRID:AB_10678258), and β-actin (Cell Signaling, 4967, RRID:AB_330288 and 3700, RRID:AB_2242334). Spectra Multicolor Broad Range Protein Ladder (Thermo Scientific) was used as a molecular weight standard. Homogenates from 4 samples were pooled together and used as an intermembrane control. Primary antibodies were diluted 1:1000 with 5% BSA in Tris-buffered saline with Tween 20 (1× Tris-buffered saline, 0.1% Tween-20). Membranes were incubated with secondary antibodies (LI-COR, Cat. no. 926-32211, RRID:AB_621843 and 926-68072, RRID:AB_10953628) diluted 1:20 000 with 5% BSA in Tris-buffered saline with Tween 20. Protein bands were detected by the Odyssey Infrared Imaging System V3.0.29 (LI-COR), and all membranes were imaged together. Band intensities were quantified using ImageJ software, and all data were normalized to β-actin protein expression and then further normalized to the intermembrane control.

### Statistical analysis

All data are presented as mean ± standard error of the mean, unless otherwise specified in the figure legends. Statistical significance level was set to α = .05, and analyses were performed using GraphPad Prism version 10 for Windows (GraphPad Software, San Diego, CA) and JMP Pro 17.0.0 (JMP Statistical Discovery LLC). Outliers were identified using the robust regression and outlier removal method (Q = 1%) and removed from the dataset prior to analysis. Data normality was determined by the Shapiro–Wilk test prior to analysis. Comparisons between active and sedentary outcomes were conducted using unpaired *t* tests or 2-way repeated measures ANOVA followed by either Bonferroni (for in vitro measures) or Tukey's (for in vivo measures) post hoc test for multiple comparisons where appropriate, and adjusted *P* values are reported. However, several comparisons (steps, MVPA, and ATP/O ratio) were analyzed by fitting with a mixed model due to missing data, which occurred due to intermittent equipment malfunction during data acquisition. Pearson correlations were used to examine the relationship between maternal in vivo and in vitro outcomes.

## Results

### Active mothers show greater aerobic fitness but similar gestational weight gain

**Figure 1:**
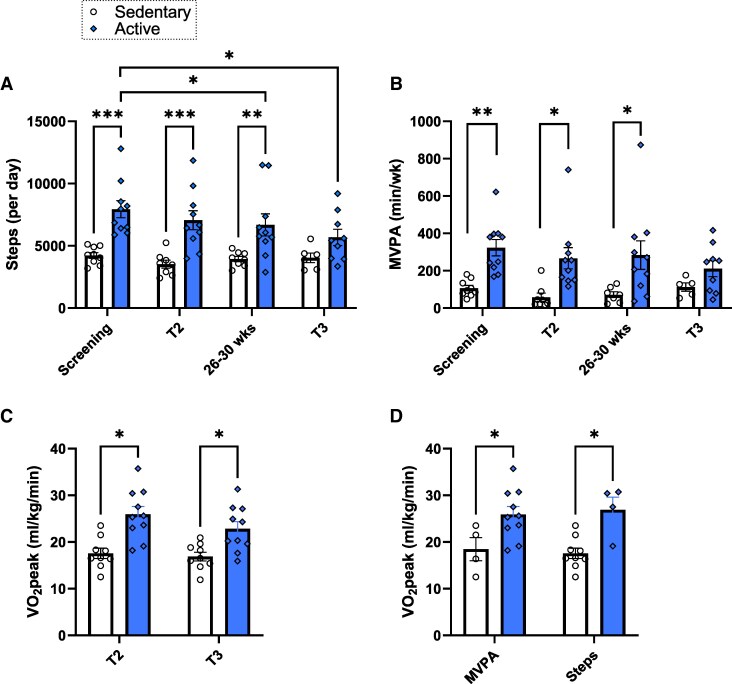
Maternal whole-body measures. (A) Daily step counts as measured by accelerometry. (B) Minutes of MVPA per week. (C) VO_2_peak measured during a submaximal treadmill exercise test. n = 9-10/group. (D) Comparison of VO_2_peak in the second trimester among participants who met the criteria for either MVPA or steps at screening. Data represented as mean ± standard error of the mean. *P < .05, **P < .01, ***P < .001.

Baseline maternal characteristics measured at screening (17.4 ± 2.7 weeks’ gestation) can be found in Table 1. Sedentary participants had an average step count of 4223.4 ± 232.7 steps per week and MVPA of 106.4 ± 14.7 minutes per week vs 7941.5 ± 688.8 steps per week and 323.3 ± 44.1 minutes of MVPA per week for active participants. As designed, the groups did not differ in age, pregravid BMI, race, or fetal sex. Bodyfat mass (37.5 ± 7.65 for sedentary mothers vs 30.58 ± 8.17% for active mothers) and total gestational weight gain (9.4 ± 4.08 for sedentary mothers vs 8.42 ± 2.88 kg for active mothers) were also not different between groups (*P* > .05). At screening and through T2, active mothers had higher daily step counts (*P* < .05), MVPA minutes per week (*P* < .01), and VO_2_peak (*P* < .05) compared with their sedentary counterparts (Fig. 1A-1C), confirming greater aerobic fitness and validating their classification as physically active. Additionally, active participants that were classified according to meeting criteria for either MVPA (*P* = .04) or steps (*P* = .003) at screening had a higher VO_2_peak in T2 compared with their sedentary counterparts (Fig. 1D). Despite a decrease in daily step counts for active mothers in T3 (*P* < .05), VO_2_peak remained higher compared with the sedentary group (22.6 ± 4.9 vs 16.9 ± 2.8 mL/kg/min, *P* < .01). Maternal diet macronutrient composition did not differ in carbohydrate, fat, protein, or total energy intake (*P* > .05) (Table 2). On average, the T2 visit occurred at 23.5 ± 1.1 weeks’ gestation, the muscle biopsy visit occurred at 28.7 ± 1.6 weeks’ gestation, and the T3 visit occurred at 33.6 ± 1.1 weeks’ gestation.

**Table 1:** Maternal baseline characteristics (<21 wks gestation)

	Sedentary (*n* = 9)	Active (*n* = 10)	*P* value
Age (years)	30 ± 4	30 ± 2	.99
Gestational age at screening (weeks)	17.7 ± 2.0	17.1 ± 3.3	.67
Gestational age at delivery (weeks)	39.3 ± 1.1	38.8 ± 1.6	.48
Race (C/AA)	8/1	10/0	.47
Height (cm)	159.74 ± 6.60	162.46 ± 6.01	.36
Weight (kg)	64.79 ± 9.11	66.25 ± 12.18	.77
Prepregnancy BMI (kg/m^2^)	24 ± 1.78	23.09 ± 3.71	.52
BMI at screening (kg/m^2^)	25.36 ± 2.84	25.1 ± 4.20	.88
Total GWG (kg)	9.4 ± 4.08	8.42 ± 2.88	.54
Systolic BP (mmHg)	99 ± 8	108 ± 6	.02*^a^*
Diastolic BP (mmHg)	60 ± 4	62 ± 4	.37
Fat mass (%)	37.50 ± 7.65	30.58 ± 8.17	.07
Fat mass (kg)	24.80 ± 7.94	21.09 ± 9.66	.38

All data expressed as mean ± SD.

Abbreviations: AA, African American; BMI, body mass index; BP, blood pressure; C, Caucasian; GWG, gestational weight gain.

^*a*^ *P* < .05, pooled *t* test

**Table 2:** Maternal diet macronutrient composition

	Sedentary (*n* = 9)	Active (*n* = 10)	*P* value
Total energy (kcal)	1413.3 ± 555.4	1525.6 ± 694.3	.70
Carbohydrates (g)	171.8 ± 477.3	162.8 ± 84.1	.81
Fats (g)	55.5 ± 27.0	65.0 ± 29.2	.47
Protein (g)	61.5 ± 25.9	79.4 ± 31.5	.20

All data expressed as mean ± SD.

### Active mothers do not display greater in vivo mitochondrial function

**Figure 2:**
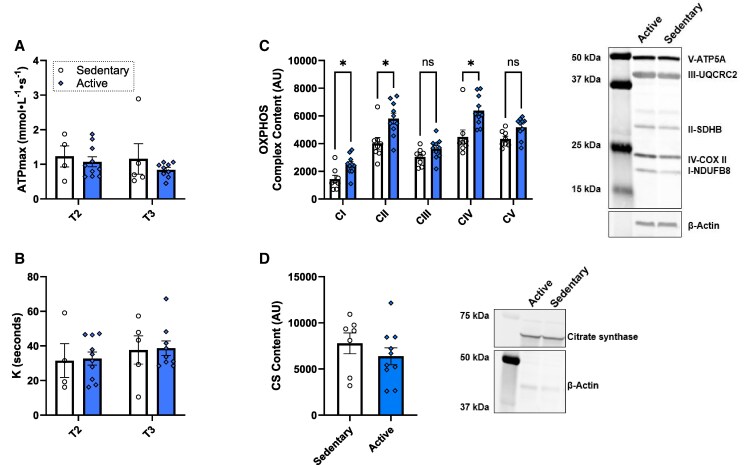
Maternal muscle measures. (A) Maximal adenosine triphosphate of the quadriceps muscle measured by ^31^P-MRS. (B) PCr τ (k) of the quadriceps muscle measured by ^31^P-MRS. (C) Oxidative phosphorylation complex content of the vastus lateralis muscle tissue, with a representative image of a Western blot. (D) CS content of the vastus lateralis muscle tissue with a representative image of a Western blot. Sedentary *n* = 4-8; active *n* = 9-10. Data represented as mean ± standard error of the mean. *P < .05. Data missing from sedentary participants at T2 (weeks 21-25; n = 5) and T3 (weeks 31-35; n = 4), and active participants at T3 (n = 1) due to curves with R^2^ < 0.7.

In vivo mitochondrial function was assessed at T2 and T3 using ^31^P-MRS. Interestingly, despite active mothers showing higher aerobic fitness, no difference was seen between groups for the maximal ATP synthesis rate (Fig. 2A) or PCr recovery time (Fig. 2B). Additionally, no differences were observed across time from T2 to T3. Interestingly, muscle tissue from active mothers showed increased protein expression of mitochondrial CI (*P* < .05), CII (*P* < .05), and CIV (*P* < .05) content, but not CIII or CV content (Fig. 2C). There was also no difference in CS (mitochondrial content marker) protein expression (*P* = .35) between groups (Fig. 2D). To further account for variability in muscle tissue mitochondrial content, the ratio of PCr τ (k) to CS and k to total OXPHOS complex content were used as markers of mitochondrial function per volume; however, there were no differences between groups (Figure S1 [38]). Additionally, no differences were observed from T2 to T3 in the active (*P*  *=* .87 and *P* = .87) or sedentary group (*P* = 1.00 and *P* = .93) for PCr τ (k) to CS or k to total OXPHOS complex content, respectively (Fig. S1B and S1D [38]). Similarly, when taking the ratio of maximal ATP (ATPmax) to CS or ATPmax to total OXPHOS complex content, no differences were observed between groups or across visits in the active (*P*  *=* .79 and *P*  *=* .66) or sedentary group (*P*  *=* .44 and *P* = .46) for ATPmax to CS or ATPmax to total OXPHOS complex content, respectively (Fig. S1A and S1C [38]).

### Active mothers do not display greater mitochondrial function in primary myotubes

**Figure 3:**
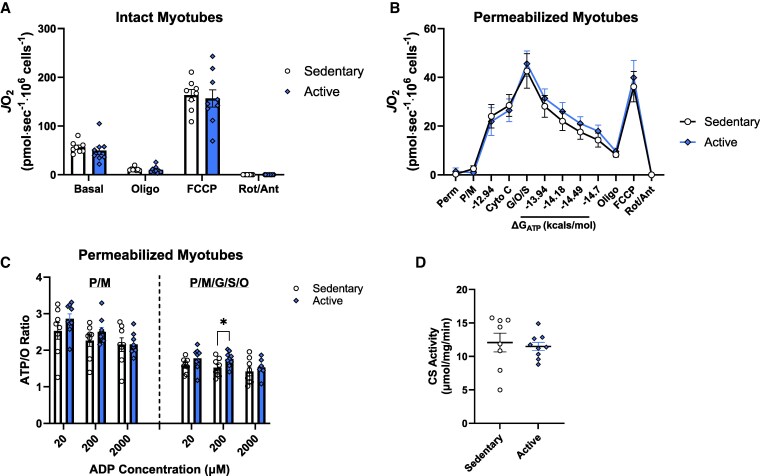
Maternal myotube mitochondrial measurements. (A) Cellular respiration of intact myotubes. The rate corresponding to the peak response from FCCP titration was used in the analysis. (B) Assessment of OXPHOS kinetics in digitonin-permeabilized myotubes. (C) ATP/O ratio with 20, 200, and 2000 µM ADP in permeabilized myotubes. (D) CS activity of myotubes. Sedentary n = 8; active n = 9. Data represented as mean ± standard error of the mean. *P < .05.

**Figure 4:**
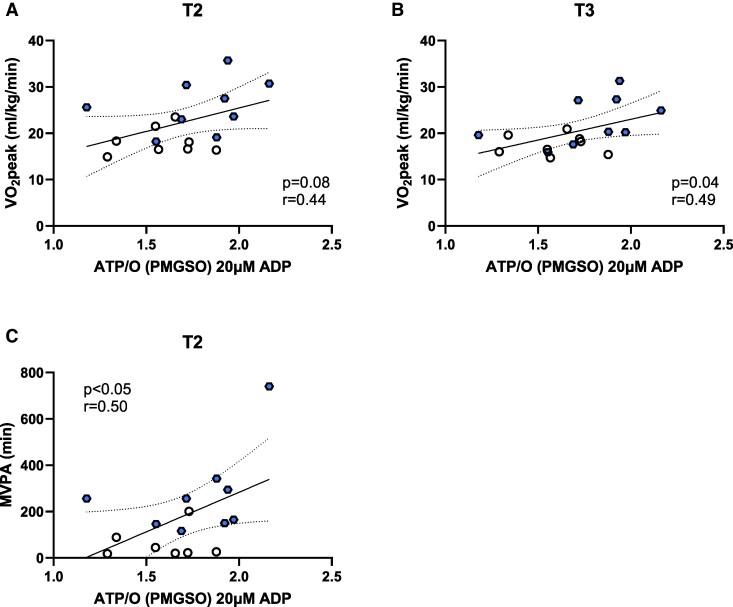
Maternal VO_2_peak correlations with myotube adenosine triphosphate/oxygen (ATP/O) ratio. (A) Maternal cardiorespiratory fitness (VO_2_peak) measured by submaximal treadmill exercise test in the second trimester correlated with myotube ATP/O with PMGSO substrates at 20 µM ADP. (B) Maternal VO_2_peak measured by submaximal treadmill exercise test in the third trimester correlated with myotube ATP/O with PMGSO substrates at 20 µM ADP. (C) Maternal MVPA in the second trimester correlated with myotube ATP/O with PMGSO substrates at 20 µM ADP. The black dotted lines represent the 95% confidence bands of the best-fit line.

In addition, in vitro mitochondrial function was assessed in SKMcs (cultured and differentiated into myotubes) obtained from a muscle biopsy taken in T3. In intact cells, no differences in *J*O_2_ were observed under basal conditions, upon ATP-synthase inhibition (Oligo), or FCCP-stimulated maximal respiratory capacity between groups (Fig. 3A). In permeabilized cells, no significant differences in *J*O_2_ were seen under any substrate condition or clamped energy demand (Fig. 3B). When taking the ATP/O ratio as an index of mitochondrial efficiency, there was no group effect, but myotubes from active mothers showed a higher ATP/O ratio with mixed substrates (P/M/G/S/O) (*P* < .05) at the 200 µM ADP concentration (Fig. 3C). However, when adjusting for multiple comparisons, significance was lost (*P* = .08). These results are in accordance with the observation of no differences in CS activity (*P* = .70; Fig. 3D). When further examining the relationship between mitochondrial function and aerobic fitness, the ATP/O ratio was seen to significantly correlate with VO_2_peak measured in at T3 (r = 0.49, *P* < .05) (Fig. 4B) and approached significance at T2 (r = 0.44, *P* = .08) (Fig. 4A). Additionally, the ATP/O ratio was positively correlated with MVPA at T2 (r = 0.50, *P* < .05) (Fig. 4C).

## Discussion

Exercise has well-documented benefits of improving insulin sensitivity [5], weight control [7], mitochondrial oxidative capacity [14], biogenesis, and function [13] and is recommended for pregnant women. The benefits of exercising during pregnancy not only extend to the mother but also to the developing offspring [39-43]. It is known that mitochondria play a pivotal role in conferring the adaptations to aerobic exercise via changes in oxidative capacity [13]. To our knowledge, there are few studies assessing muscle physiology during pregnancy. Thus, the purpose of this study was to compare mitochondrial bioenergetics between active and sedentary expectant mothers and evaluate changes across T2 and T3. The overall observations indicate no differences with in vivo or in vitro mitochondrial respiratory capacity or efficiency between active and sedentary mothers, but there is an overall relationship between aerobic fitness and mitochondrial efficiency. Taken together, this may suggest that the hallmark mitochondrial adaptations that occur with physical activity, such as increased mitochondrial respiratory capacity, content, and efficiency, may not be directly transposable in the context of pregnancy.

In this study, active mothers had higher physical activity compared with the sedentary group through 26 to 30 weeks of gestation as measured by accelerometry. We saw a decline in physical activity in active mothers throughout T2 and T3, a well-documented pattern in pregnancy [1]. Regardless of this decrease, active mothers maintained a higher VO_2_peak compared with sedentary mothers through T3, indicating maintenance of aerobic fitness levels. Despite differences in aerobic fitness, active and sedentary mothers had no difference in maximal ATP synthesis rate or PCr recovery time. ^31^P-MRS measurements were taken in T2 and T3 where physiological insulin resistance is heightened, and this advanced insulin resistance may play a role in reduced mitochondrial capacity [8, 10, 44]. Differences in ^31^P-MRS measurements between active and sedentary nongravid populations can be teased out when rates are corrected for differences in mitochondrial content [14]. However, in our groups, we saw no difference in mitochondrial ^31^P-MRS measurements when normalized to mitochondrial or OXPHOS content, suggesting that neither mitochondrial nor OXPHOS content is a primary contributor to mitochondrial function for this population. Increases in aerobic fitness may stem from other physiological adaptations. For example, exercise-related improvements in aerobic fitness and mitochondrial efficiency can be driven by a multitude of factors such as changes in the proteome, metabolome, or even nonskeletal muscle adaptations [45]. Considering our active mothers had increased protein content of mitochondrial complexes I, II, and IV but not overall increased mitochondrial content, this supports the notion that exercise-related improvements in aerobic fitness can be partly driven by intrinsic adaptations in the electron-transport system. Further, pregnancy-related changes, such as increased blood volume and vascularity, may also increase measures of aerobic fitness [46]. Together, this suggests that aerobic fitness may not necessarily correlate with changes in mitochondrial function during pregnancy, or the activity level may have been inadequate to elicit differences in mitochondrial content or function [47].

As an in vitro method of examining mitochondrial efficiency, the ATP/O ratio was quantified by simultaneous measures of ATP production and O_2_ consumption. There was no difference between groups when fueled by CI-linked substrates; however, myotubes from active mothers showed a higher ATP/O ratio at 200 µM when energized by mixed substrates (both CI- and CII-linked). Significance at the 200 µM level with mixed substrates may show that compared with sedentary mothers, active mothers have increased efficiency under certain energy demands, but this difference was lost when accounting for multiple comparisons. ATP/O was positively correlated with MVPA at the start of T2 (r = 0.50, *P* < .05; data not shown), consistent with higher physical activity levels during early pregnancy. Additionally, during T3, the ATP/O ratio was found to correlate with VO_2_peak, aligning with the well-documented relationship between higher fitness levels and enhanced mitochondrial efficiency as an adaptation to aerobic exercise [48-50]. Therefore, even though there were no group differences in in vitro mitochondrial measures, they were well-aligned with maternal fitness throughout pregnancy.

No differences in in vitro mitochondrial respiratory capacity were observed in intact or permeabilized cells between the groups. These findings are contrary to an analogous study in nonpregnant women. Heden et al observed a 38% higher maximal oxygen consumption in permeabilized SKMcs in aerobically trained women compared with their untrained counterparts [17]. Our findings may differ because the muscle biopsy used for in vitro experiments was taken in T3 where time spent in physical activity was declining for active mothers, making them comparable to their sedentary counterparts. Additionally, the Heden et al study's active participants had a higher VO_2_peak compared with our active group, and our study did not consider the intensity of exercise when recruiting active participants, contrary to Heden et al. A number of studies have shown that exercise-induced changes in mitochondrial respiratory capacity only occur with higher training volumes and intensities [51-53], thus reinforcing that the training stimulus may have been inadequate in our active participants to elicit changes in mitochondrial function. This may be especially true due to our study being observational and focused on recreationally active individuals who became pregnant.

We next looked at mitochondrial content differences between the groups. CI, CII, and CIV were higher in active mothers compared with sedentary mothers, with CIII and CV trending to be higher in active mothers. Exercise has been shown to increase electron transport proteins in trained individuals [54, 55]. However, in a study comparing mitochondrial protein expression in pregnant women with and without GDM, they discovered no differences in mitochondrial complex protein content, despite lower activity of CI, CIII, and CIV in mothers with GDM [8]. Additionally, mitochondrial supercomplexes have been shown to form as a result of aerobic exercise to increase overall efficiency of electron transfer and to reduce the production of reactive oxygen species [56]. In a study of healthy men and women, CI to CIV had the potential to exist as part of super complexes after 16 weeks of aerobic exercise training [55]. This could explain the reason for increased mitochondrial efficiency with increased levels of fitness without a corresponding increase in total mitochondrial content. This leaves an area for future exploration in mitochondrial adaptations during pregnancy.

It is important to note that this study is not without its limits. Maternal blood samples could have provided additional insights regarding the potential benefits of exercise during pregnancy. Aerobic physical activity was examined in this study; therefore, these results cannot be extrapolated to expectant mothers who partake in other modalities of exercise. Moreover, in vitro experiments utilized skeletal muscle taken during T3 where active mothers’ physical activity was beginning to decline, leading to the possibility of loss of mitochondrial adaptations during this time. Future studies should take biopsies over the time course of pregnancy to explore these changes. Additionally, the cross-sectional nature of this study does not allow us to determine a causal relationship between maternal exercise and the bioenergetic measures reported in this study. However, the novel approach of this study should be noted. We are, to our knowledge, the first to explore the muscle physiology and mitochondrial bioenergetics of expectant mothers in vivo with ^31^P-MRS and in vitro in primary skeletal muscle myotubes. Therefore, an additional strength is the pairing of in vitro and in vivo measures of mitochondrial bioenergetics during pregnancy.

In conclusion, this study revealed that in active mothers with higher physical activity and aerobic fitness, no differences in in vivo or in vitro mitochondrial capacity existed when compared with their sedentary counterparts, albeit some protein contents of the electron transport chain were higher in active women. This implies that physiological changes in late gestation may influence how regular exercise impacts mitochondrial bioenergetics. However, confirmation of this effect would require a longitudinal study throughout pregnancy. The reduction in physical activity throughout T2 and T3 that was seen in the active group may explain these findings. Due to the findings that aerobic fitness and MVPA in early pregnancy correlate with in vitro mitochondrial efficiency, recommendations should emphasize aerobic training strategies to maintain mitochondrial outcomes throughout pregnancy.

## Data Availability

Data are available on request from the corresponding author.

## Clinical trial information

Trial registration number ClinicalTrials.gov Identifier: NCT03489564.
